# A generic error-related potential classifier based on simulated subjects

**DOI:** 10.3389/fnhum.2024.1390714

**Published:** 2024-07-17

**Authors:** Aline Xavier Fidêncio, Christian Klaes, Ioannis Iossifidis

**Affiliations:** ^1^Faculty of Electrical Engineering and Information Technology, Ruhr University Bochum, Bochum, Germany; ^2^Robotics and BCI Laboratory, Institute of Computer Science, Ruhr West University of Applied Sciences, Mülheim an der Ruhr, Germany; ^3^KlaesLab, Department of Neurosurgery, University Hospital Knappschaftskrankenhaus, Ruhr University Bochum, Bochum, Germany

**Keywords:** error-related potential (ErrP), adaptive brain-machine (computer) interface, generic decoder, ErrP classifier, EEG, SEREEGA, simulation

## Abstract

Error-related potentials (ErrPs) are brain signals known to be generated as a reaction to erroneous events. Several works have shown that not only self-made errors but also mistakes generated by external agents can elicit such event-related potentials. The possibility of reliably measuring ErrPs through non-invasive techniques has increased the interest in the brain-computer interface (BCI) community in using such signals to improve performance, for example, by performing error correction. Extensive calibration sessions are typically necessary to gather sufficient trials for training subject-specific ErrP classifiers. This procedure is not only time-consuming but also boresome for participants. In this paper, we explore the effectiveness of ErrPs in closed-loop systems, emphasizing their dependency on precise single-trial classification. To guarantee the presence of an ErrPs signal in the data we employ and to ensure that the parameters defining ErrPs are systematically varied, we utilize the open-source toolbox SEREEGA for data simulation. We generated training instances and evaluated the performance of the generic classifier on both simulated and real-world datasets, proposing a promising alternative to conventional calibration techniques. Results show that a generic support vector machine classifier reaches balanced accuracies of 72.9%, 62.7%, 71.0%, and 70.8% on each validation dataset. While performing similarly to a leave-one-subject-out approach for error class detection, the proposed classifier shows promising generalization across different datasets and subjects without further adaptation. Moreover, by utilizing SEREEGA, we can systematically adjust parameters to accommodate the variability in the ErrP, facilitating the systematic validation of closed-loop setups. Furthermore, our objective is to develop a universal ErrP classifier that captures the signal's variability, enabling it to determine the presence or absence of an ErrP in real EEG data.

## 1 Introduction

Brain-computer interfaces (BCIs) are systems developed to allow communication between the brain and external devices. They are widely applied for the rehabilitation or assistance of patients suffering from motor impairments caused, for example, by an amputation, spinal cord injury, or stroke (Soekadar et al., 2015; Abiri et al., 2019; Kumar et al., 2019). Due to its good temporal resolution, reduced cost, and usability, electroencephalography (EEG) is currently the most popular technique used for recording neural activity in the development of non-invasive BCIs (Kumar et al., 2019).

Based on the recorded EEG data, different brain signals types can be decoded and used to control an external device (e.g., a prosthetic arm). The specification of the experimental paradigm to use will define which mental task the BCI user is supposed to perform and which kind of stimulus is to be presented by the BCI to elicit the brain signals of interest. Common BCI paradigms are based on event-related synchronization/desynchronization (ERS/ERD), steady-state visual evoked potentials (SSVEPs), or P300 potentials (Abiri et al., 2019). The decoding of brain activity is, however, still a challenging task, and optimal performance is usually not achieved.

To improve their BCIs, recent works have proposed using a specific neural activity signal generated during performance monitoring and referred to as error-related potentials (ErrPs) (Xavier Fidêncio et al., 2022). ErrPs are the neural signature of error processing in the brain, elicited not only by self-made errors but also error made by an external system. Different BCI paradigms can be used to elicit them (for a review, see Chavarriaga et al., 2014; Kumar et al., 2019; Xavier Fidêncio et al., 2022), and these signals can be used, for example, to correct a mistake made by the interface.

Seven different types of ErrPs are usually reported in the BCI literature. Self-made errors are called *response ErrPs* (Blankertz et al., 2002; van Schie et al., 2004), *feedback ErrPs* inform about the outcome of a choice (Miltner et al., 1997; Chavarriaga et al., 2014) and *target ErrPs* reflect the response to unexpected changes in the task (Diedrichsen, 2005). Most common in BCI are *interaction ErrPs* (Ferrez and Millán, 2005, 2008b), evoked when the system misinterprets the user's intention, and *observation ErrPs*, which are generated while the subject observes a system over which they have no control making a mistake. Lastly, *execution and outcome ErrPs* have also been reported, reflecting unexpected movements (Diedrichsen, 2005; Spüler and Niethammer, 2015) or undesired outcomes (Krigolson et al., 2008; Spüler and Niethammer, 2015; Kreilinger et al., 2016), respectively. For a comprehensive review of the taxonomy and protocols used for each error type, please refer to Xavier Fidêncio et al. (2022).

A current limitation in applying ErrPs to improve BCIs involves their accurate classification on a single-trial basis. The implication is that experimental protocols need to include a calibration phase to collect a representative number of error trials to train a subject-specific ErrP classifier. Only after training can this ErrP classifier be applied online to provide feedback to the BCI. These calibration sessions are time-consuming, usually between 20 and 30 min, and might be too monotonous and tiring for the subject. Existing results applying transfer learning and leave-one-subject-out approaches suggest that a generic ErrP classifier can be used to remove or at least reduce the calibration time for new subjects. However, a decrease in performance is still present when compared to the baseline classifier trained for each subject. Therefore, this work contributes to the existing literature toward a generic subject-independent ErrP classifier.

We show the proof-of-concept of a generic error-related potential classifier training framework. As (Iturrate et al., 2011; Schonleitner et al., 2019), we expect that a generic model trained with more subjects would better transfer to a new dataset as the model could learn more subject-independent features while focusing less on subject-specific characteristics, which would improve generalization performance. We further hypothesize that, by using a simulated dataset that meaningfully represents real-data variabilities and deviations, it is possible to create a generic ErrP classifier that generalizes to unseen real individuals with high performance. Using simulated data for training allows flexibility on the number of trials and subjects without limitations that can occur in experiments with real participants, such as tiredness and lack of focus. Additionally, the applied toolbox allows systematic parameter changes to account for variability in the ErrP generation, enabling a systematic evaluation of the classification framework before deployment and establishing boundary conditions for a successful closed-loop application.

The rest of this work is as follows: Section 2 reviews studies that have proposed generic ErrP classifier or other strategies to reduce calibration time. In sequence, we present the proposed generic classifier training framework and describe the datasets used in this study in Section 3. Section 4 presents our results. Finally, we conclude this work with a brief discussion and overview in Section 5.

## 2 Related work

To the best of our knowledge, only a few works have evaluated a generic ErrP classifier to reduce or eliminate the calibration phase. Iturrate et al. (2011) first proposed reducing the calibration time by using inter-subject information. By applying a leave-one-subject-out strategy, a Linear Discriminant Analysis (LDA) was trained with the data of three subjects and tested in the remaining one. The data in the interval [0.2, 0.8] s extracted from eight fronto-central channels was first normalized and decorrelated (using principal component analysis), and the *r*^2^ coefficient was applied for feature selection. The trained subject-independent classifier achieved an average accuracy of 70% over all subjects. For comparison, a baseline decoder was trained using the decimated data from channels FCz and Cz only and achieved 76% classification accuracy. Their study also analyzed an adaptive calibration approach starting with an initial generic classifier and updating each class mean based on the newly labeled data from the testing subject. This approach was tested using the same features as the baseline decoder, and they report that it can be useful for some part of the training, as accuracies stagnated after more trials were added.

In their follow-up work, the same authors attempted to reduce the calibration time by applying a transfer between different tasks instead. They proposed a latency correction algorithm to remove variations in ERP latency between two experiments, and results show that it is possible to train an observation ErrP classifier in the data from a previous experiment, reducing calibration time and with accuracies around 81.01% (Iturrate et al., 2012). However, correcting for the delay in the ERP latencies between two experiments was required, as only the between-tasks transfer always performed worse than a baseline classifier (Iturrate et al., 2014). Iturrate et al. (2013) also shows that an inter-task transfer significantly reduces the ErrP detection accuracy. They also applied the supervised adaptation strategy as proposed by Iturrate et al. (2011), and it improved the classifier transfer as more trials from the target class were used, but it did not outperform the baseline classifier trained in the target data only.

Kim and Kirchner (2016) tested a classifier transfer between different error types within the same subject to reduce calibration time. They used observation ErrPs for decoder calibration and interaction ErrPs for testing, which allowed the collection of more training trials. After spatial-temporal filtering using xDAWN (Rivet et al., 2009) and time-domain features extraction, a linear Support Vector Machine (SVM) classifier was trained. The classifier achieved a balanced accuracy of 79% across all subjects, but for four (out of eight) subjects, the transferred classifier performed worse than a subject-specific classifier. Performance was, however, better than when applying a leave-one-subject-out strategy with interaction ErrP data for training (75%).

Bhattacharyya et al. (2017) proposed an ensemble of an LDA, a quadratic discriminant analysis (QDA), and a logistic regression (LR) as a generic ErrP classifier. The features for the classifier were based on the filtered, baseline-corrected, downsampled, and normalized data from 35 selected channels and task-related features (such as session and feedback number). The generic classifier trained on the data of 16 subjects and tested on 10 independent subjects achieved an accuracy of 73.97% and an f1-score of 83.53%.

A generic shrinkage LDA (rLDA) classifier for the asynchronous ErrP detection was proposed by Lopes-Dias et al. (2019, 2020). They recorded data from 15 subjects and applied a leave-one-subject-out approach based on time-domain features. They found no significant differences in the comparison between the generic and a subject-specific rLDA in terms of true-positive and true-negative rates (subject-specific: *TPR* = 70.0%, *TNR* = 86.8% and generic: *TPR* = 72.6%, *TNR* = 87.9%). They also observed that, while for some above-average subjects, the generic classifier performed worse, for below-average ones, classification performance was even higher than when using the subject-specific classifier.

Lastly, Schonleitner et al. (2019) also proposed a generic rLDA classifier trained on available data from other subjects. They performed a comparative study between a generic model and its adapted version using both supervised and unsupervised strategies against the subject-specific decoders. The generic classifier was obtained by averaging the parameters of 100 rLDA models trained on a balanced dataset, obtained after downsampling the majority class. Time-domain features given by signal mean in ten partially overlapping windows were used for classification. For their experimental data from 12 subjects, the subject-specific classifier achieved a balanced accuracy of 88.3%, and the generic classifier showed an inferior performance (72.7%). As expected, their supervised adaptation shows that using labeled trials from the unseen subject can significantly improve the generic classifier accuracy and achieve results almost equivalent to the optimal subject-specific decoder (84.3%). The problem with this approach is the need for labeled ErrP trials of the new subject. On the other hand, the unsupervised adaptation of the generic classifier led to an overall worse result in their analysis (68.5%), only working for some subjects. Further analyses indicated, however, that unsupervised adaption can be beneficial if the initial general model accuracy is above the mean accuracy found (>72.7%). This reduced performance is also understandable, considering that the label used for classifier adaptation was dependent on the current adapted model accuracy, which is uncertain.

## 3 Materials and methods

The classification of ErrPs involves distinguishing between the error and the correct conditions on a single-trial basis. We propose a novel training framework based on simulated subjects as an attempt to remove the calibration phase required to train an ErrP classifier. Our hypothesis is that, as data simulation allows us the flexibility to generate a large dataset for training with less effort (more trials and subjects), if the effects of the error-related neural activity can be realistically simulated, then a classifier trained in this dataset should be able to capture the relevant features for this classification task, such that for new unseen real-subject, it would perform well, with no or little performance loss. Details on the framework implemented are described in the following sections.

### 3.1 Simulated ErrP datasets

**Figure 1:**
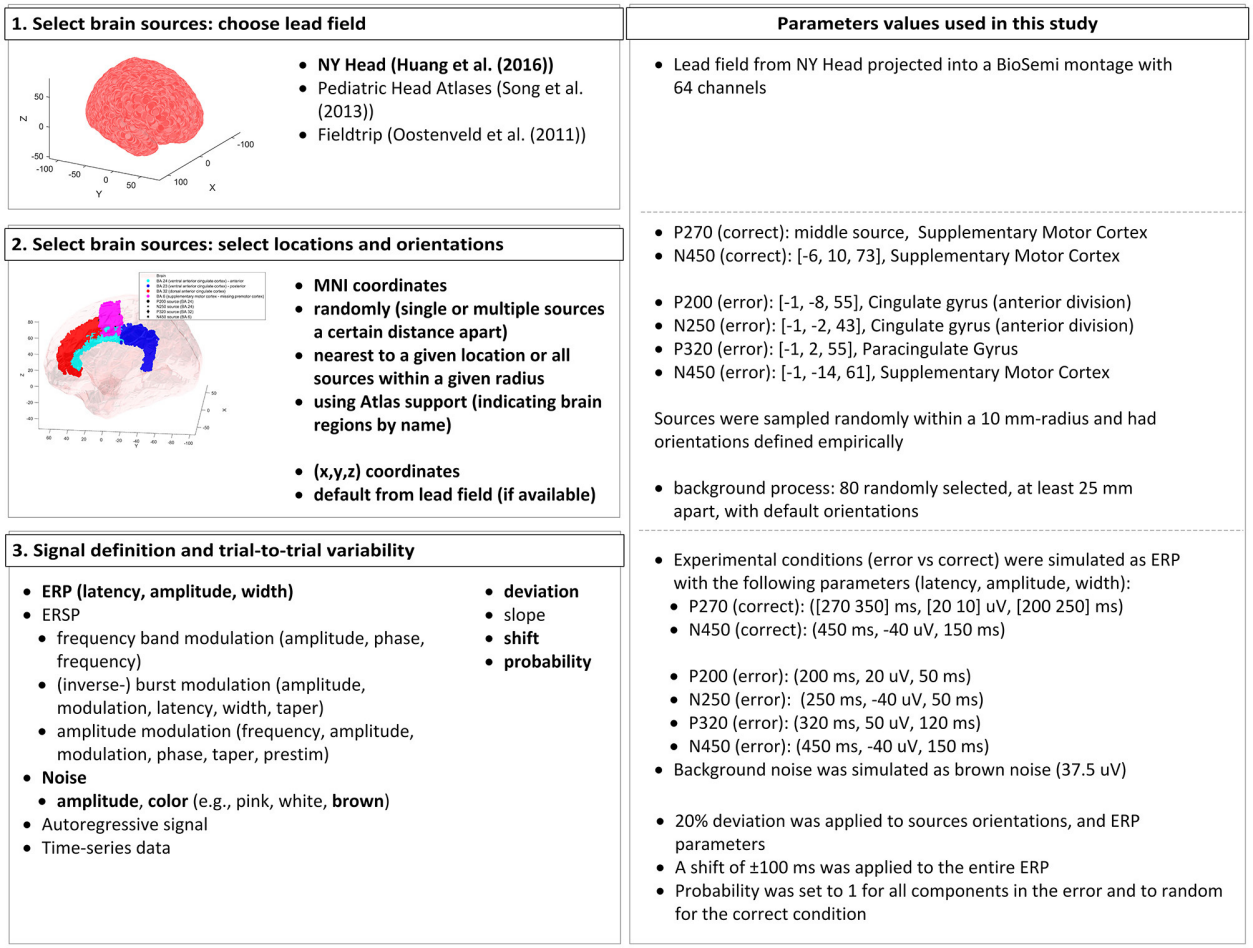
Summary of the parameters used in the SEREEGA toolbox to generate the simulated ErrP dataset. **(Left)** The workflow and list of options the toolbox offers, highlighting in bold the features used in this study. **(Right)** We summarize the parameter values used in this study.

To demonstrate the feasibility of the proposed classifier training approach, we simulated an interaction ErrP, considering that this error type is common in BCI applications. The EEG data was simulated using the open-source toolbox SEREEGA (Krol et al., 2018, version 1.5.0^1^), which allows the simulation of event-related epochs with known ground truth. The EEG data simulation is based on the forward problem of EEG: starting with a forward model, we can define brain sources with different activation patterns, and by projecting these activation patterns onto the scalp surface, the EEG data is generated (for details, please refer to Krol et al., 2018). An overview of the parameters used and the available options in the toolbox can be seen in Figure 1.

In SEREEGA, it is necessary to simulate each experimental condition separately. Therefore, to simulate ErrP data, the error and correct conditions were generated individually. During human-machine-interaction, the error condition exhibits the components: P200-N250-P320-N450 (Ferrez and Millán, 2005, 2008b, 2009; Omedes et al., 2015; Spüler and Niethammer, 2015). The correct condition was simulated as a P270-N250-complex (Ferrez and Millán, 2005; Omedes et al., 2015; Spüler and Niethammer, 2015).

The first step required is to select brain sources from a head model. We used the New York Head Model (Huang et al., 2016) lead field with 74.382 available sources. This lead field includes default orientations and projection parameters that indicate how an activation at a given source is projected onto each electrode. The toolbox allows sources to be obtained based on exact MNI coordinates, randomly or by indicating specific regions in the brain. We selected the sources' centers for each component based on reported locations (Ferrez and Millán, 2008b; Omedes et al., 2015; Spüler and Niethammer, 2015). The sources' orientations were defined empirically by inspection of the projection patterns generated from randomly chosen orientations. The orientations generating similar topography as reported by Ferrez and Millán (2008b, 2005, 2009) were selected, and a ±20% deviation was randomly applied to produce variability across subjects.

The experimental conditions (error vs. correct) were simulated as ERP classes. The error condition has evoked potentials centered around 200, 250, 320, and 450 ms, with amplitudes of 20, −40, 50, and −40 μV (at source level), and widths of 50, 50, 120, and 150 ms, after the findings from Ferrez and Millán (2008b) and Omedes et al. (2015). For the correct condition, the negative peak is centered at 450 ms, with amplitude −40 μV and width 150 ms. The positive components consist of two peaks to simulate the slower, longer effect visible in the grand average used as reference (inspired by the P3b component from Krol et al. (2018). The peaks are centered at 270 and 350 ms, with widths of 200 and 250 ms and amplitudes of 20 and 10 μV. Considering that only a few papers have reported the correct condition grand average, and not all of them report these ERP components, the probability of each peak being generated was set randomly. For all other ERP components, the probability was always set to 1, meaning the peak is generated in every trial.

To simulate variability across trials, for each subject, all parameters (latency, width, and amplitude) were given a deviation of 20% of their value, as done by Krol et al. (2018). Moreover, a random latency shift of ±100 ms was applied to the entire ERP to simulate shifts in the ERP latencies while keeping its overall shape. Brown noise was also projected from 80 randomly selected sources, at least 25 mm apart from each other, with an amplitude of 37.5 ± 0.5 μV to simulate background processes (Kobler et al., 2019). In summary, the values for the trial-to-trial variability parameters were kept consistent across subjects, but each trial was generated independently. Therefore, the parameter values for each trial were sampled within their respective ranges, considering the original values and the variations defined by the parameters.

For all simulations, the data was projected onto the pre-defined BioSemi montage with 64 channels. The sampling frequency was set to 250 Hz, and epochs were 1.5-s long, including a pre-stimulus period of 500 ms. In total, 1, 200 epochs were simulated per subject, with an error rate of 20%, as is common in ErrP protocols. For the training dataset, 15 subjects were simulated. An independent validation dataset included 10 simulated subjects. To simulate inter-subject variabilities, for each subject simulated, the source locations were randomly sampled around the defined center in a radius of 10 mm. Considering SEREEGA's default behavior in its current release, the simulation of a single dataset with several trials is not equivalent to simulating multiple subjects. Therefore, to achieve the mentioned spatial variabilities, we simulated multiple subjects. Additionally, this approach also closely resemble real-life datasets structures, in which data from several subjects are included.

### 3.2 Real-life ErrP datasets

The trained classifier was also validated with real subject data. We used the data we collected in two studies. The experiments were not originally designed for this study, but as they contain ErrP data, it is particularly suitable to use them. The studies involving human participants were reviewed and approved by the Ethics Committee of Medical Faculty of the Ruhr University Bochum. The participants provided their written informed consent to participate in the studies.

#### 3.2.1 Experimental protocol

**Figure 2:**
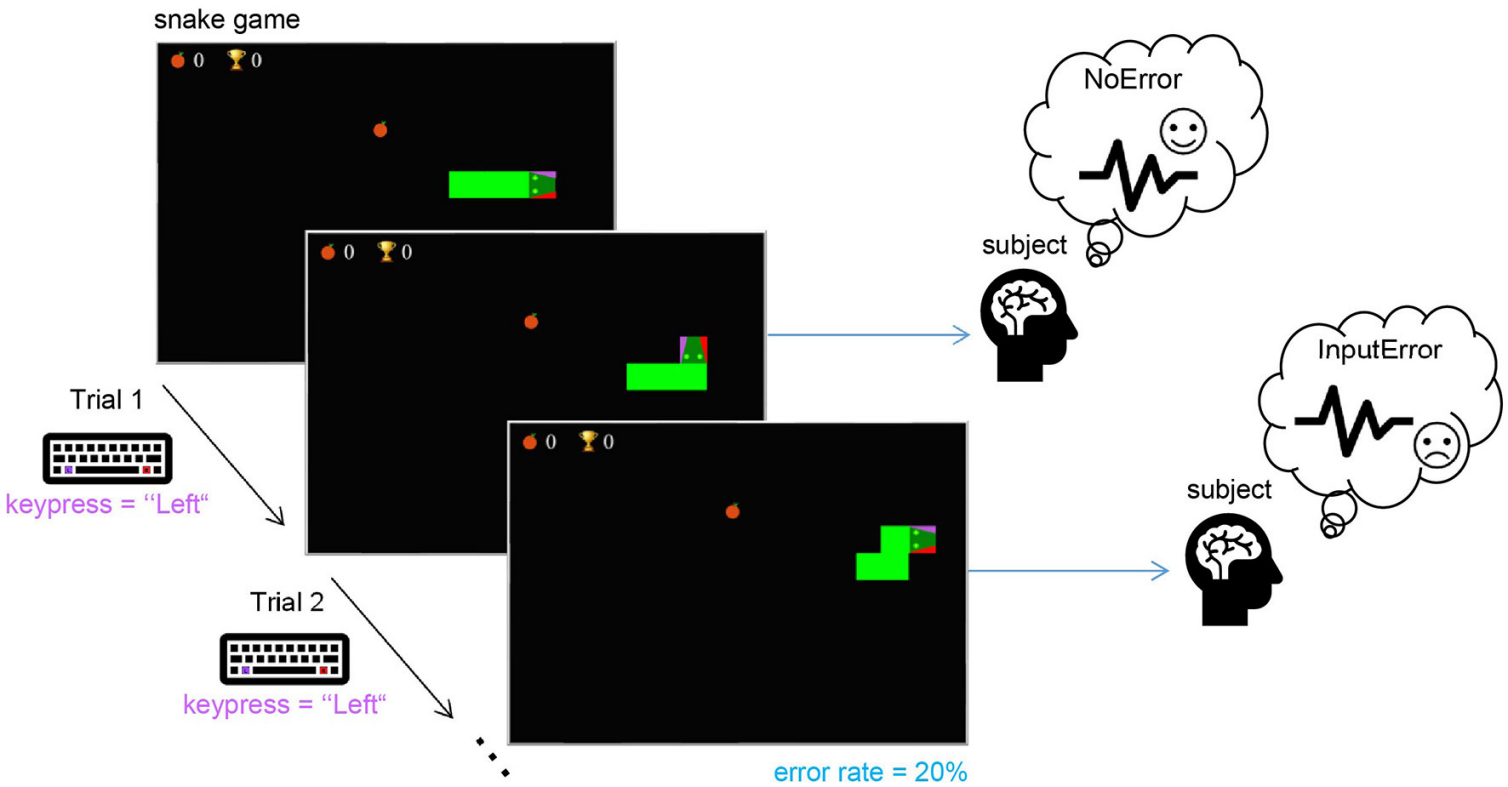
The experimental task used for the real-life data: the subject played the game by pressing either the “Alt” (for left) or “Altgr” (for right) keys on the keyboard to control the snake's direction to avoid collision with itself and collect as many points as possible. In each trial, if the subject pressed a key to change the current movement direction of the snake, with a probability of 20%, the snake moved in the wrong direction (as depicted in Trial 2) to elicit an ErrP.

The first real-life dataset (dataset 1) used contains the data collected in a study designed for interaction ErrP detection using a simple task. The subjects played a modified snake game with a keyboard (see Figure 2). Each subject was instructed to use left- and right-hand index fingers to press two keys on the keyboard (“Alt” and “Altgr,” respectively) in order to interact with the game and control the snake's movement. As in the original version, the snake moves forward with a certain speed (constant, in this study), it cannot move backward, and the goal is to collect as many points as possible. The player has two options to change the current movement direction: either to the left or right (w.r.t. the current moving direction). In this task, each trial is given by the keypress. To elicit ErrP, a fixed error rate of 20% was used. The subjects played the game without errors during a familiarization phase to avoid self-made errors in the data. When subjects were confident about the key mapping, they were allowed to play a few trials with an active error rate to see what the real task would look like. Subjects were instructed to observe whether the snake moved in the direction they wanted.

Ten healthy subjects (five male, age: 28.6 ± 6.0), all right-handed with normal or corrected-to-normal vision participated in this study. The data from two subjects had to be excluded due to technical issues during recording. Additionally, the data from one subject was excluded because they did not have proper sleep the night before and reported being extremely tired and unfocused during the recording. Therefore, the data of seven subjects (four male, age: 26.6 ± 4.9) was analyzed in this study.

The second real-life dataset (dataset 2) includes the data recorded while subjects played a modified version of the snake game shown in Figure 2 adapted for motor imagery-based control. The experimental protocol is described in the Supplementary material. Interaction errors were artificially introduced with a low rate of 5% to keep subjects motivated, since the primary goal of this study was the collection of motor imagery data, not ErrPs. The data from four subjects were not included in this study. Details can be found in Appendix A. Therefore, in this study we used the data from 13 healthy subjects (six male, age: 25.6 ± 3.9), all right-handed, with normal or corrected-to-normal vision to validate the generic classifier.

#### 3.2.2 Data recording

EEG was recorded using two different systems to compare their results in dataset 1. A dry Brain Products Xpress Twist EEG system with 32 active electrodes (FP1, Fz, F3, F7, FT9, FC5, FC1, C3, T7, FPz, CP5, CP1, Pz, P3, P7, O1, Oz, O2, P4, P8, FCz, CP6, CP2, Cz, C4, T8, FT10, FC6, FC2, F4, F8, FP2) was used in session 1. Data was recorded with a sampling rate of 1000 Hz, and GND/REF electrodes were placed behind the ears. Session 2 used a Neuroelectrics Enobio EEG system with 8 electrodes (FC1, FC2, C3, Cz, C4, CP1, CP2, Pz), sampled at 500 Hz and CMS/DRL placed behind the right ear. Each subject participated in two recording sessions performed on the same day, first wearing the dry and then the wet EEG system, and 1200 trials were recorded per session. The session order was chosen considering that after using the wet EEG system the hair had to be rinsed. Exceptionally, subjects 4 and 7 needed their sessions to be recorded on different days. Details about the experimental protocol for headset setup are described in Supplementary material. In the dataset 2 recordings were done with the wet EEG.

#### 3.2.3 Data analysis

Data was preprocessed using EEGLAB (Delorme and Makeig, 2004), and the following steps were applied to each dataset, as commonly used in the ErrP literature (Ehrlich and Cheng, 2016): (1) Downsampling to 250 Hz. (2) Notch and band-pass filtering using a zero-phase Hamming windowed sinc FIR filter with cutoff frequencies of 1 and 20 Hz. (3) Automatic artifact channel rejection and interpolation using the clean_rawdata and ICLabel (Pion-Tonachini et al., 2019) plugins in EEGLAB, and (4) Re-referencing to common average (CAR).

The data was further segmented around the movement onset of the snake after a key press. This is the time when the user receives feedback on whether it moved in the desired direction. Epochs were extracted for the interval [−0.2, 0.6] s around the feedback onset. Epochs containing automatic snake movements or collisions with itself were excluded from further analyses. Table 1 summarizes the events kept for each subject in each dataset.

**Table 1:** Summary of the number of correct (c) and error (e) trials included in the real-life ErrP datasets for each of the subjects analyzed (*n* = 7 for dataset 1 and *n* = 13 for dataset 2).

		**S01**	**S02**	**S04**	**S05**	**S06**	**S07**	**S10**							**Avg ±std**
Dry	e	214	204	226	199	235	231	235							220.6 ± 13.8
	c	962	965	961	977	951	940	948							957.7 ± 11.4
Wet	e	229	230	236	218	226	203	250							227.4 ± 13.5
	c	957	941	956	952	957	865	929							936.7 ± 30.8
		**S01**	**S02**	**S03**	**S04**	**S05**	**S06**	**S07**	**S08**	**S09**	**S10**	**S11**	**S12**	**S13**	
MI + ErrP	e	35	32	32	44	13	30	29	21	29	24	21	39	22	28.5 ± 8.3
	c	789	876	683	659	539	742	650	652	552	537	518	679	566	*649.4 ± 108*

### 3.3 Single-trial ErrP classification

For the single-trial ErrP classification, we applied commonly used methods for feature extraction and classification as proof-of-concept. As time-domain features are widely applied for the classification of ErrPs, we computed the mean amplitude in eight overlapping time slots as temporal features: [0, 100], [100, 200], [150, 250], [200, 300], [250, 350], [300, 400], [350, 450], [400, 500] ms w.r.t. the time-locking event. As the wet EEG system used for the real-life dataset only has eight channels, the features were calculated only for those.

The works reviewed in Section 2 have shown the feasibility of using simple classifiers such as LDAs or SVMs for generic ErrP detection. Therefore, an SVM classifier with a radial kernel was applied as a proof-of-concept. Features were normalized over all trials and used to train the classifier. Considering the unbalanced nature of the ErrP classification, we used class weights as inversely proportional to the class frequencies (Kim and Kirchner, 2016). The cost (i.e., regularization) and gamma parameters of the SVM were optimized using Optuna (Akiba et al., 2019) and predetermined values for the search: [10^−6^, 10^−5^, …, 10^6^] and [10^−5^, 10^−4^, …, 10^5^], respectively. The balanced accuracy (arithmetic mean of true positive and true negative rates) was used as a metric for classifier performance evaluation and hyperparameter optimization. For classifier evaluation, 10-fold cross-validation was applied. To account for the class imbalance, the majority class was randomly downsampled in the training samples. Testing was performed on the imbalanced splits. Given the random sampling, the cross-validation was repeated 10 times, and the results are reported as the overall mean.

After training, the generic ErrP classifier was applied to each validation dataset (simulated, dry, wet, and MI + ErrP), and no further parameter tuning was done. The classifier performance is reported for each unseen validation subject and compared to a subject-specific classifier and a classifier trained using the leave-one-subject-out approach. For dataset 2, as only very few error trials were recorded for each subject, no subject-specific classifier was trained.

## 4 Results

### 4.1 Neurophysiological analysis of the real-life ErrPs

**Figure 3:**
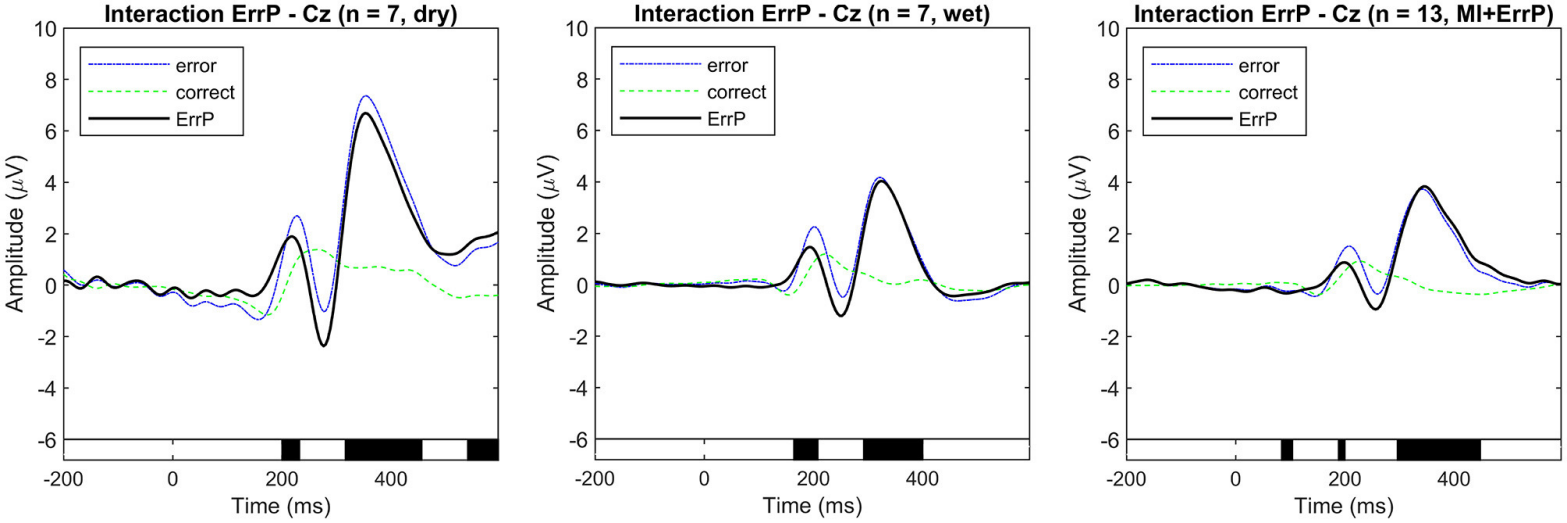
Event-related potentials at electrode Cz were averaged over all subjects for each condition (error and correct). ErrPs are given as the difference grand average (error minus correct) for each EEG type used for recording (dry and wet EEG systems) in real-life dataset 1. On the right, the ErrP from the motor imagery and ErrP study (real-life dataset 2). On the bottom, the black background shows the time intervals with a significant difference between error and correct trials (*p* < 0.05; corrected for multiple comparisons by false discovery rate (FDR) to avoid false positives). Results are consistent with related works.

The overall grand average event-related potentials for the correct and error conditions are shown in Figure 3 for the real-life data recorded using the dry and wet EEG systems at channel Cz. This channel was chosen following standard practice in the error data analysis, considering that ErrPs show a fronto-central distribution, with higher amplitudes expected at channels FCz and Cz (Xavier Fidêncio et al., 2022). Baseline correction was applied using the pre-stimulus interval [−0.2, 0] s, and the ErrPs are given as the difference: error minus correct. For the dry EEG, the ErrP ERP shows the following components: a positive peak at 216 ms, a negative peak at 276 ms, a positive peak at 252 ms, and a small negative peak at 508 ms. For the wet EEG: positive peak at 192 ms, a negative peak at 252 ms, a positive peak at 324 ms, and a small negative peak at 456 ms. Significant differences between error and correct trials are present in both cases. The observed ErrPs present waveform shape consistent with other studies (Ferrez and Millán, 2005, 2008a,b, 2009).

**Figure 4:**
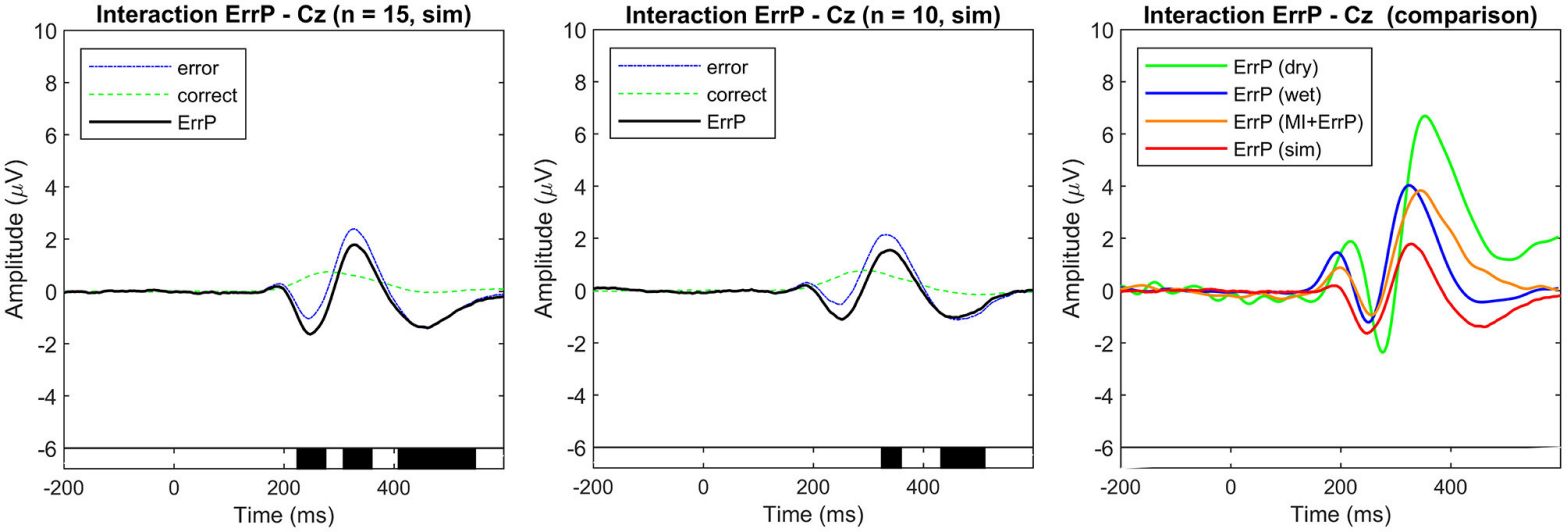
Event-related potentials at electrode Cz were averaged over all simulated subjects for each condition (error and correct). ErrPs are given as the difference grand average (error minus correct). **(Left)** Simulated dataset used for generic ErrP classifier training (*n* = 15). **(Center)** Simulated dataset used for classifier evaluation (*n* = 10). On the bottom the black background shows the time intervals with a significant difference between error and correct trials (*p* < 0.05; corrected for multiple comparisons by false discovery rate (FDR) to avoid false positives). The simulated ErrP shows ERP components as expected and according to the literature used as a reference for the simulation parameters. **(Right)** Comparison of ErrPs across datasets (dry, wet, MI + ErrP, and simulated training data).

As shown in Figures 3, 4, there are visible latency and amplitude differences between the two ErrPs (dry vs. wet). A one-way within-subjects ANOVA was performed to compare the ErrP measured with the two EEG systems. We computed two separate ANOVAs for peak amplitudes and latencies. For each subject, the most prominent peaks in the following intervals were selected: positive peaks at [50, 250] ms and [200, 400] ms and negative peaks at [200, 400] ms and [400, 600] ms. We used the python library *pingouin* (Vallat, 2018) and configured it to compute Mauchly's test of sphericity and correct *p*-values using Greenhouse-Geisser when applicable, as also done by Iturrate et al. (2012) and Kim and Kirchner (2016) while performing the same kind of ERP comparison. Statistically significant differences were found only for the P200 latency [*F*_(1, 6)_ = 14.008, *p* = 0.010], the N400 amplitude [*F*_(1, 6)_ = 40.030, *p* = 0.001] and both amplitude [*F*_(1, 6)_ = 17.171, *p* = 0.006] and latency [$F_{\left(1,6\right)}=174.519,p<1e^{-4}$] of the P300 component. Whether these differences are related to the EEG devices or the generated error-related activity in each session would need to be further investigated, but this falls out of the scope of this work. For the purpose of evaluating the proposed generic classifier, both datasets can be applied. Figure 3 also shows the interaction ErrP collected with a lower error rate during the motor imagery-based control of the snake game. The measured interaction ErrPs are consistent with related works.

### 4.2 Neurophysiological analysis of the simulated ErrPs

**Figure 5:**
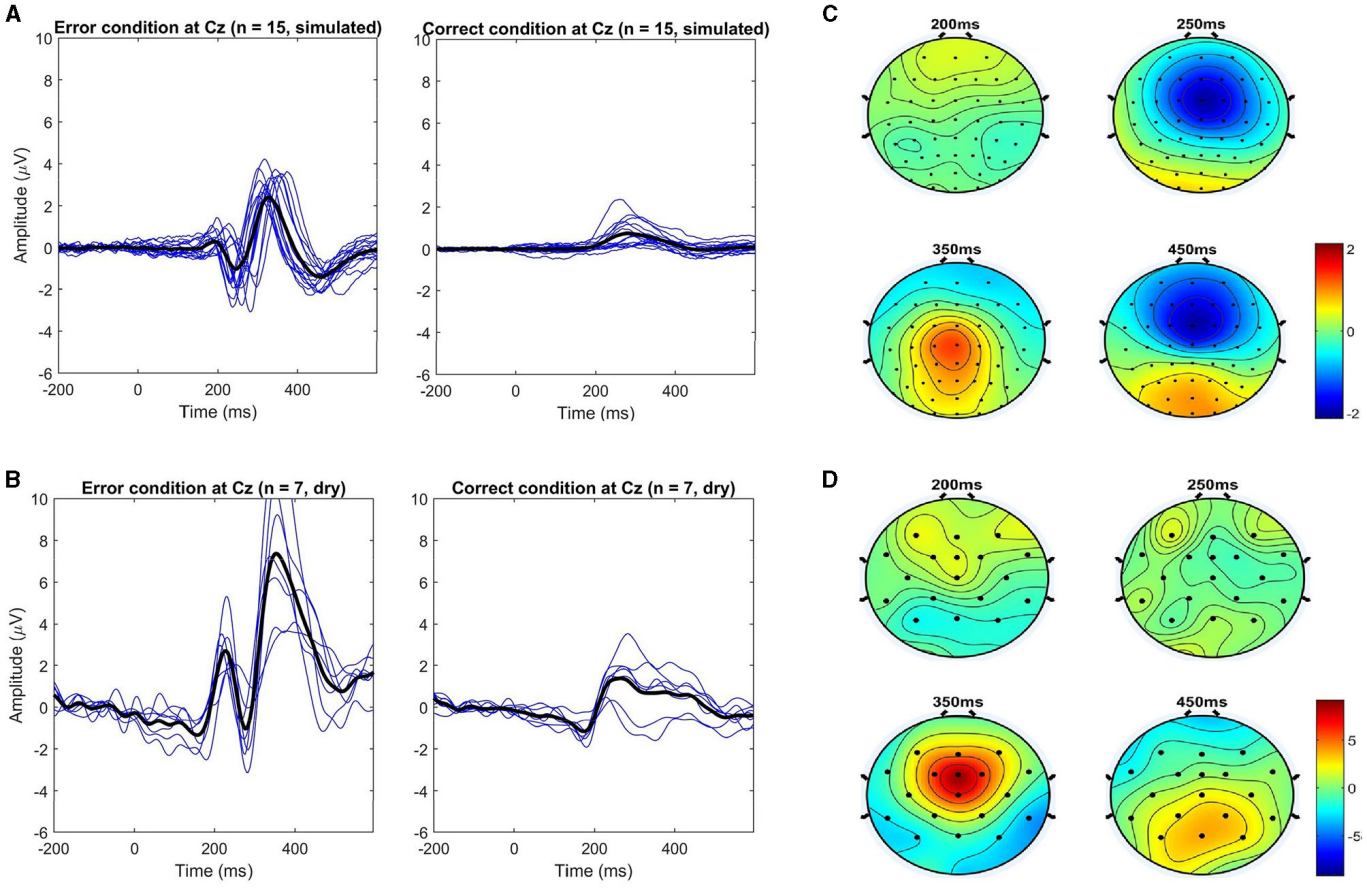
Comparison of the event-related potentials at electrode Cz. **(A, B)** show each condition separately (error vs. correct) for each subject (blue lines) and the overall average (black lines) for both simulated and dry datasets, respectively. **(C, D)** show the respective topographical distributions of the error minus correct (ErrP) at the most prominent peaks. Note that all channels were used (64 for the simulated, and 32 for the dry EEG data).

The overall grand averages for the simulated datasets are shown in Figure 4. The observed ErrPs present waveform shape consistent with the literature and reference results used for simulation parameters choice, with positive peaks at 184 and 328 ms and negative peaks at 248 and 460 ms for the simulated training dataset with similar values for the simulated validation dataset. Figure 5 shows the ERPs for each condition and subject for the simulated data used for training and the real-life data measured with the dry EEG system for comparison. It is possible to see the inter-subjects' variabilities in both datasets and expected similarities between simulated and real data.

The topographic maps for the respective ErrPs shown in Figure 5 highlight the expected frontal-central location of the error-related activity in both datasets, especially for the positive peak around 350 ms. For the negative peaks, the spatial location is more clearly visible in the simulated data. However, a closer look reveals that in both cases, as time passes, parietal areas become more active, like around the last negativity at 450 ms, as expected (Ferrez and Millán, 2008b).

### 4.3 Single-trial ErrP classification

**Figure 6:**
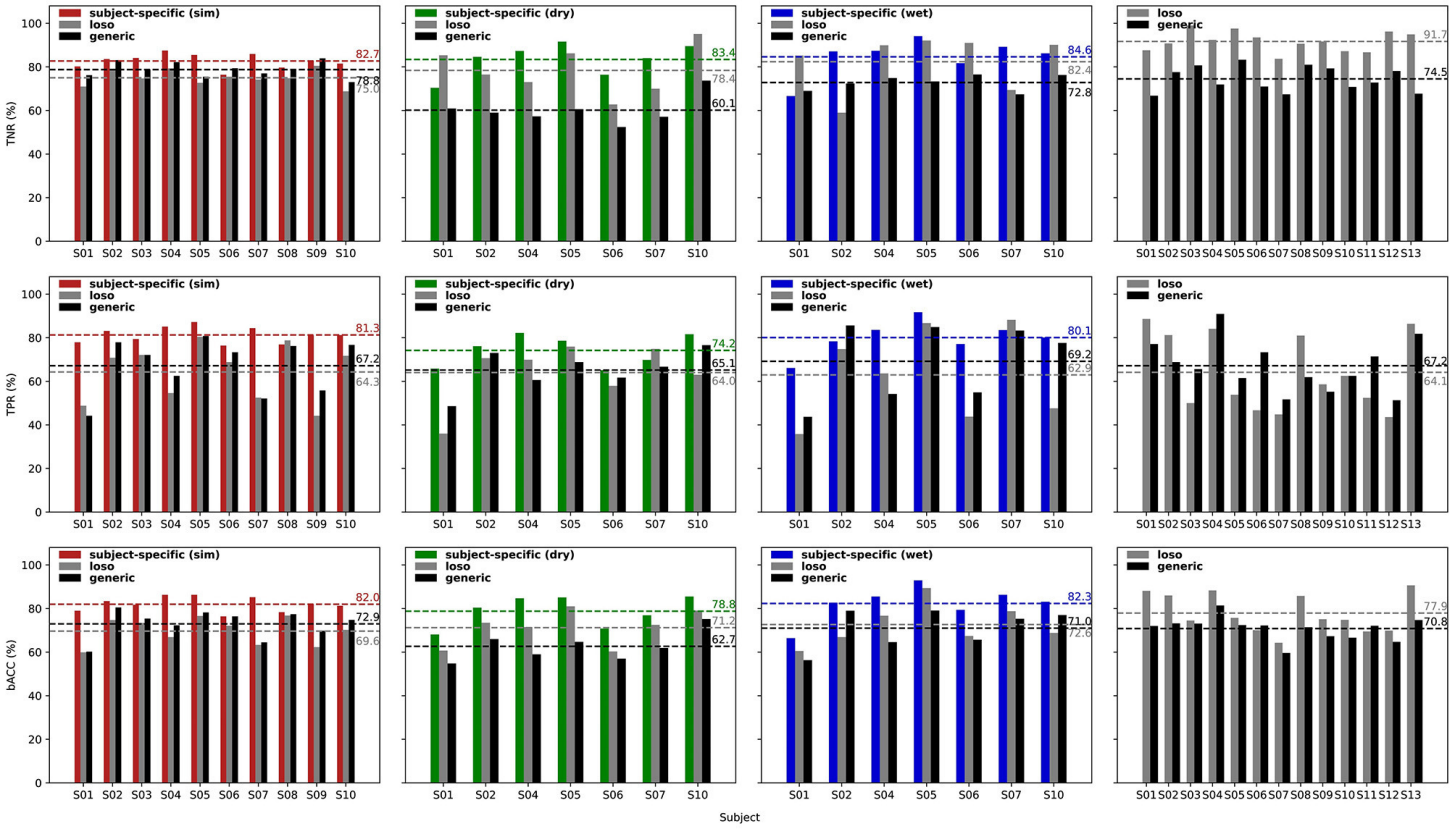
Classification performance for the subject-specific, leave-one-subject-out, and generic classifiers obtained for each dataset used for validation: simulated (left, red plot), dry EEG (middle, green plot), wet EEG (middle, blue plot), and MI + ERRP (right plot) datasets. As expected, the subject-specific classifiers show the highest performance for all datasets. Note that for the MI + ErrP no subject-specific classifier was trained because the error rate was only 5%. However, the generic classifier shows promising generalization across datasets and subjects. While it performs worse than the subject-specific models, its accuracies are similar to the loso approach for the error detection. Note that the error class is the positive class (TPR, true positive rate; TNR, true negative rate; bACC, balanced accuracy). All classification results are significantly above the chance level (*p* < 0.05).

For each validation dataset, the performance and comparison between a subject-specific, a leave-one-subject-out, and the generic ErrP classifier can be seen in Figure 6.

The subject-specific classifier was trained as a baseline for comparison, and the parameters were also optimized using Optuna (Akiba et al., 2019). The results obtained with a 10-times 10-fold cross-validation are reported for the simulated validation and the real-life datasets, respectively. The significance of the classification results was evaluated using a cross-validated permutation test (1,000 permutations), and all results reported are significantly above chance level (*p* < 0.05). We also compared the accuracies against results obtained with a classifier trained using a leave-one-subject-out (loso) as described in the works listed in Section 2. In this case, for each testing subject, the data from the other subjects were used for training.

On average, the subject-specific classifier achieved a balanced accuracy of 82.0% for the simulated data, 78.8% for the dry EEG data, and 82.3% for the wet EEG data, and the generic classifier shows balanced accuracies of 72.9%, 62.7%, and 71.0%, respectively. These results are similar to what has been reported in the literature: while applying a generic classifier for the ErrP detection seems possible, it does not outperform a subject-specific classifier trained with that subject's data directly.

**Figure 7:**
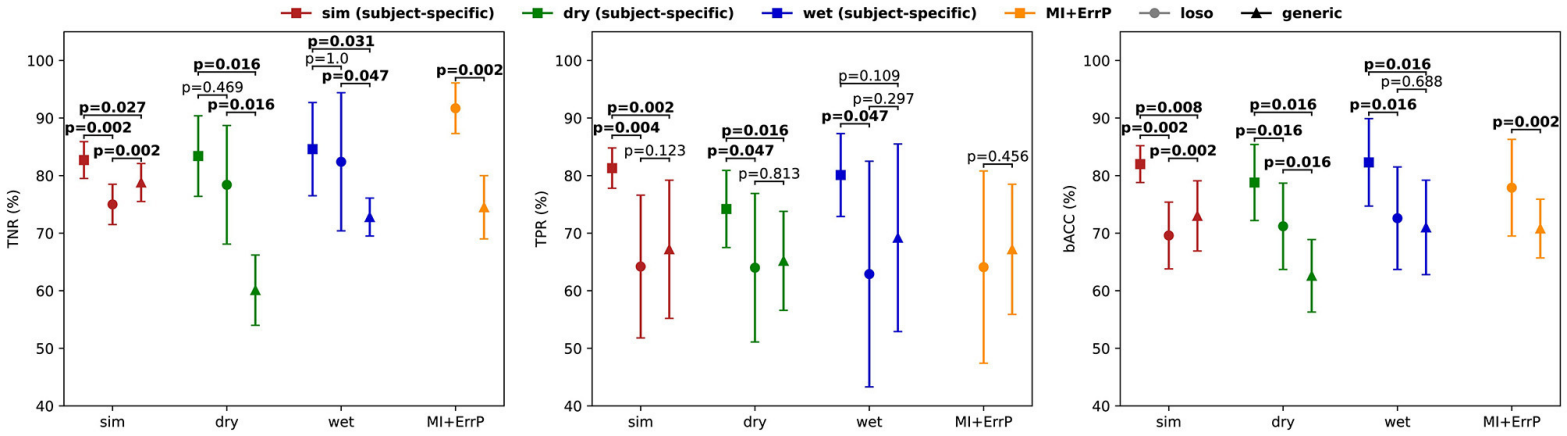
Performance comparison between generic, leave-one-subject-out (loso), and subject-specific classifiers in terms of TNR, TPR, and bACC (± STD). Significant test results of a two-sided Wilcoxon signed rank test are shown for each dataset.

To test if the differences in the results are significant, we performed a two-sided Wilcoxon signed rank test for both TPR and TNR for each validation dataset. Results are shown in Figure 7 and indicate that the classifier performances were significantly different, except for the error class classification in the wet EEG data (*p* = 0.109). We also tested whether the generic classifier was worse than the subject-specific using one-sided tests. Results indicate that the generic classifier was significantly worse for all datasets and metrics (sim: *p* = 0.014 for TNR, *p* < 0.001 for TPR; dry: *p* = 0.008 for both TPR and TNR; wet: *p* = 0.016 for TNR), except again for the error class in the wet EEG data (*p* = 0.055 for TPR).

The leave-one-subject-out classifier (loso) achieved balanced accuracies of 69.6% for the simulated testing data, 71.2% for the dry dataset, 72.6% for the wet dataset, and 77.9% for the MI + ErrP experiment dataset. The generic classifier performs similar to it in the simulated data for TPR (*p* = 0.062, one-sided test for generic better than loso). This was expected, since both are based on simulated data and on a leave-one-subject-out approach. Surprisingly, the generic classifier performed significantly better for TNR (*p* < 0.001, one-sided test). Our hypothesis is that using more subjects for training contributed to the better generalization. The comparison between the loso and the generic classifier on the real-life data also shows similar performance for TPR for dry (*p* = 0.656, one-sided test for generic worse than loso), wet (*p* = 0.891, one-sided test for generic worse than loso), and MI + ErrP (*p* = 0.795, one-sided test for generic worse than loso) datasets. This is a very relevant result, as it indicates that replacing real-subject with simulated data for the generic classifier training is possible for the error classification. On the other hand, the generic classifier is significantly worse for TNR, also for both datasets (*p* = 0.008 for the dry, *p* = 0.023 for the wet, and *p* < 0.001 for the MI + ErrP dataset). This lower performance for the correct trials can be a direct result of the parameters used for the correct class simulation. Defining these parameters were more difficult than for the error class, since only few works report the ERP components and respective possible source location. If more is known about the signal generation, the simulation could be adapted accordingly and classification results could be improved.

Overall, the single-trial classification results show that the generic classifier performs significantly worse than the subject-specific for all datasets tested. This result is in line with related work described in Section 2, which systematically report that using a generic classifier is possible, but it implies a reduced performance. On the other hand, when compared to a leave-one-subject-out approach using the data from other subjects, the generic classifier shows similar performance for the error detection. This highly promising result validates our hypothesis that utilizing simulated data can effectively decrease calibration time by substituting real subjects typically employed for training the generic classifier.

Despite the visible high inter-subject variability in performance, the generic classifier proposed shows some promising generalization across different datasets and subjects, considering that no further training or adaptation was applied. Individual differences and their impact on classifier performance and generalization need investigation. A deeper understanding of the intra-subject variability and single-trial characteristics of the ErrPs can also help to tailor generic classifiers with better performance.

## 5 Discussion

This paper introduces a novel framework for training a generic error-related potential (ErrP) classifier, focusing on the interaction ErrPs crucial for Brain-Computer Interfaces (BCIs). The study employs a Support Vector Machine (SVM) classifier for single-trial classification, utilizing simulated EEG data generated with SEREEGA and real-life data from a simplified snake game task. The results indicate that the generic classifier performs effectively across various datasets, although its performance, for now, is not as high as that of subject-specific classifiers.

The simulation parameters were based on well-established literature results regarding the so-called interaction ErrPs (Ferrez and Millán, 2005, 2008b, 2009; Omedes et al., 2015; Spüler and Niethammer, 2015). This ErrP type is particularly relevant for BCIs because, as it is generated while the BCI user perceives a mistake made by the interface while interacting with it, it can be used to improve its performance, for example, by error correction or to drive error-based learning. For the single-trial classification of ErrPs, a Support Vector Machine (SVM) classifier based on time-domain features was used as a proof-of-concept, as related work has already shown the feasibility of using such methods for the error classification.

The generic classifier was validated using unseen simulated and real-life data and compared to a subject-specific classifier trained on these datasets. Ten independent subjects not seen during training were simulated for validation. Two real-life datasets were used to validate the classifier performance. In both experiments, subjects played a simplified version of the snake game. Dataset 1 contains the data from ten subjects, recorded in two sessions using different EEG devices for their comparison. Dataset 2 contains the data from 13 subjects collected while they played an adapted version of the snake game for motor imagery-based control (MI + ErrP).

Results show that the generic classifier is able to reach balanced accuracies of 72.9%, 62.7%, 71.0%, and 70.8% on the simulated data, the data recorded with a dry EEG system, the data recorded with a wet EEG system, and the MI + ErrP experiment data, respectively (Figure 6). Despite the visible high inter-subject variability in performance, the generic classifier proposed shows some promising generalization across different datasets and subjects, considering that no further training or adaptation was applied. Compared to the subject-specific classifiers (balanced accuracies of 82.0%, 78.8%, and 82.3%, respectively), the proposed generic classifier performed significantly worse. This decrease in performance is in line with findings from related works that have proposed generic classifiers based on leave-one-subject-out approaches (see results reviewed in Section 2). A leave-one-subject-out approach applied to the validation datasets achieved balanced accuracies of 69.6% for the simulated testing data, 71.2% for the dry dataset, 72.6% for the wet dataset, and 77.9% for the MI + ErrP dataset. The generic classifier performed similarly to it for error detection (TPR) but significantly worse for the correct trials classification (TNR). This lower performance for the correct class can be explained by the parameters used for the correct class simulation, which were more difficult to define since only a few works report the ERP components and respective possible source locations. As more about the signal generation becomes known, the simulation could be adapted accordingly, and classification results will likely improve. The advantage of the proposed framework is that the simulated data frees all subjects from a monotonous and exhausting calibration phase and provides a ready-to-use decoder. In addition, SEREEGA enables the systematic change of simulation parameters to account for variabilities in ErrP signal generation, including the probability of whether a signal should be generated. On closed-loop frameworks using ErrPs for improvement, this allows their systematic validation and establishing boundaries for their usage. However, even though using generic classifiers for ErrP detection seems possible, they did not achieve optimal results. Some aspects can be considered to improve the performance of a generic ErrP classifier based on simulated data.

Firstly, a deeper understanding of the inter-subject variability and single-trial characteristics of the ErrPs is of key importance to developing classifiers with higher performance. ErrPs are commonly reported in terms of the difference grand average (error minus correct) over all trials and subjects, if grand averages are, at all, reported (Xavier Fidêncio et al., 2022). While this approach provides information about the ERP shape of the ErrP signal, it masks the inter-trial and between-subjects variabilities, which is relevant for their single-trial-basis classification. Even fewer studies provide additional analysis, such as topographical scalp distribution and source localization. Additionally, it is also not very common to perform the frequency-domain analysis of the ErrP. This frequency information could be explored in the future, not only for frequency-based features for classification but also to adapt the simulation parameters and include these modulations.

**Figure 8:**
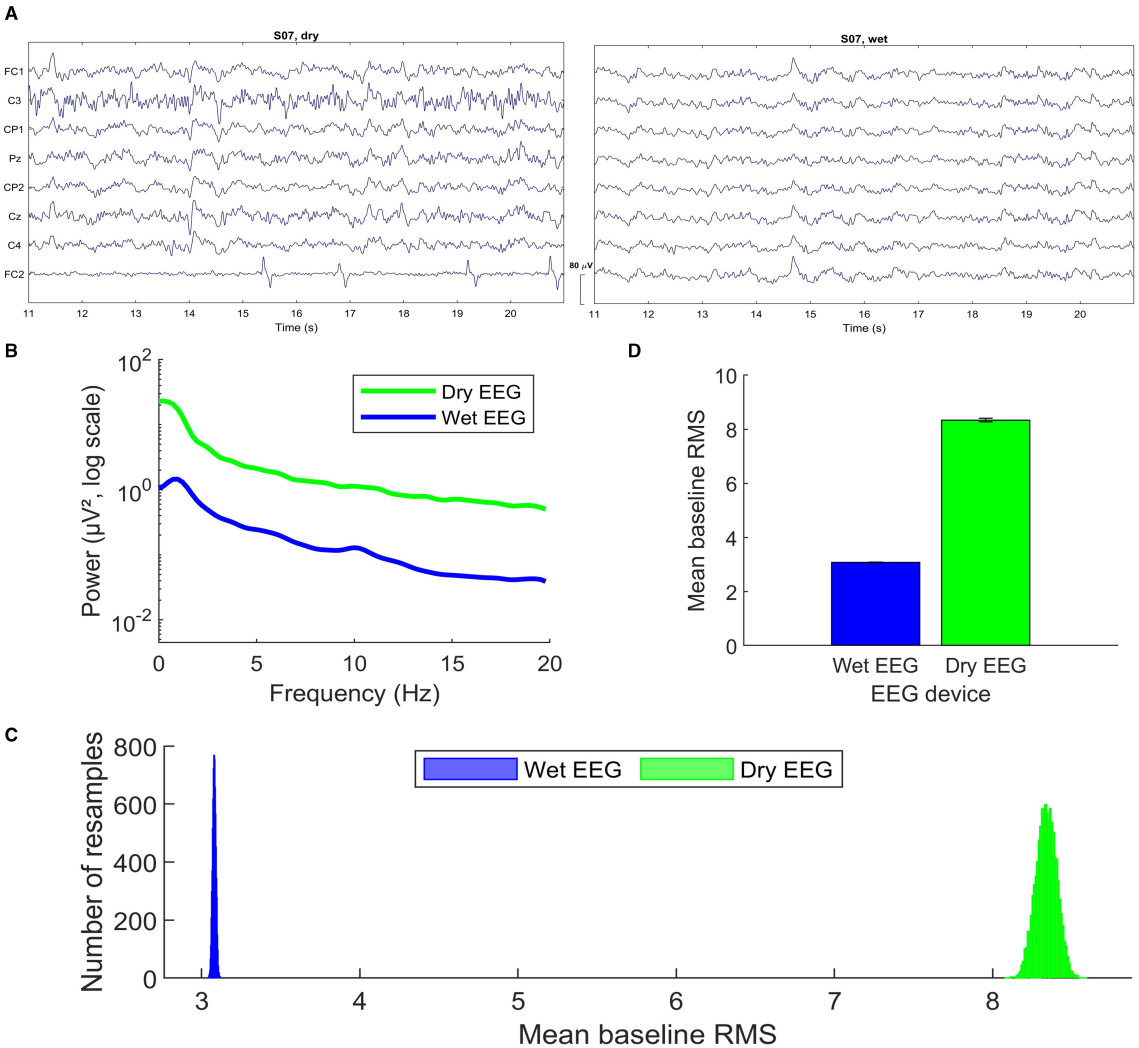
Dry and wet EEG signal quality (dataset 1): **(A)** Raw EEG data (notch-filtered) recorded with each headset for one subject. Only the common channels across systems are depicted, using the same scale and time interval with respect to the start of the recording. **(B)** Single-trial spectra for channel Cz computed using Fast Fourier Transform on 575 correct trials averaged over trials and subjects. **(C)** Histogram of the grand average root mean square (RMS) values during the 200 ms baseline before feedback onset, for 10.000 permutations of 575 randomly selected correct trials. Values are averaged over electrodes, trials, and subjects, in this order. **(D)** Mean of the permuted distributions shown in C. Figures **(B–D)** were produced following the methods described by Mathewson et al. (2017) to perform the comparison between dry and wet EEG devices.

The analysis of the data measured with two different recording devices (dry and wet EEG systems) showed some significant differences in the observed ErrPs for the same subjects in terms of peak latency and amplitude. Figure 8A shows the notch-filtered raw data recorded for one subject with both headsets, as an example. Following the methods described by Mathewson et al. (2017), we calculated the grand average frequency spectra over all subjects. For each subject, Fast Fourier Transform was applied to 575 randomly selected no-error (i.e., correct) epochs at channel Cz and averaged to compute the participant's spectra (Figure 8B). Both spectra show the expected 1/f-like decay. As observed by Mathewson et al. (2017), there is a broad-band power increase for the dry EEG system. The amplitudes are consistently higher across all frequency bands when compared to the wet system, which is an indicator of higher overall EEG signal amplitudes captured by the dry electrodes. This observed behavior can often suggest potentially more noise in the data, possibly due to higher electrode impedance or less optimal skin-electrode contact inherent to dry electrodes. These observations can also explain the decreased classification accuracies for the data recorded with the dry EEG, despite the observed higher amplitudes and the use of time-domain features.

A second estimate of the noise in the data recorded with each EEG system was computed with the root mean square (RMS) for a 200 ms-baseline period before the feedback onset. The RMS can be used as an estimate of the single-trial noise in EEG data because it measures the average absolute difference of the voltage around the baseline (Mathewson et al., 2017). To estimate the distribution of the RMS values, 10.000 permutations were applied such that RMS values were calculated for 575 randomly sampled correct condition epochs, averaged over electrodes, trials, and subjects, in this order. Figure 8C shows the histogram of the grand average single-trial RMS values computed. The mean and standard deviation of the RMS of each distribution is plotted in Figure 8D. The results show a clear difference between each RMS distribution. The dry EEG system (*mean*_*rms*_ = 8.3396;*std*_*rms*_ = 0.0659) shows larger single-trial noise levels when compared to the wet EEG (*mean*_*rms*_ = 3.0799;*std*_*rms*_ = 0.0105).

**Figure 9:**
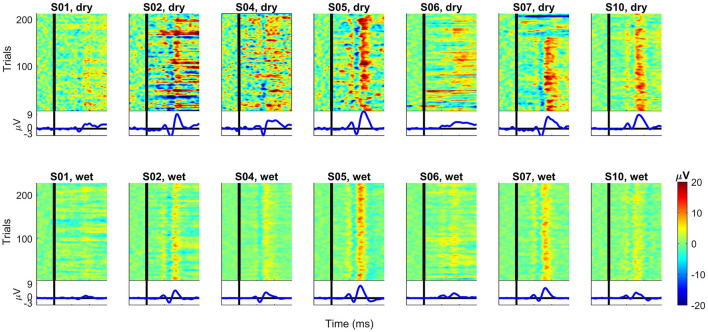
ERP images for each subject and recording EEG devices at channel Cz. For each subject, the average over all correct trials were subtracted from each error trial. After smoothing over five trials, the data was color-coded and combined into one image. First trial is at the bottom and last trial is at the top of each plot. For comparison, all plots have the same scale. The vertical black lines denote the time *t* = 0 ms (feedback onset).

Figure 9 shows the ERP images for each subject and recording system. For each subject, we subtracted the average over all correct trials from each error trial for channel Cz. The data was smoothed over five consecutive trials for random noise reduction while keeping the single-trial dynamics of the error signal. The resulting potentials were then color-coded and stacked to construct the final ERP image. In this case, it shows trials in their original order of appearance during task performance (bottom to top). Below each image is the average ERP over the plotted trials.

The individual ERP traces in Figure 9 show that the ErrP waves resemble the overall grand averages (see Figure 3) for most subjects. For subject S06 (dry), the overall ErrP does not clearly show the expected positive and negative components in the waveshape compared to other subjects (note that the data have the same scale). The recording with the wet EEG system for the same subject S06 shows lower amplitudes, but the ErrP shape looks more similar to the others. Another exception is subject S01, for whom not even with the wet EEG, a clear ErrP with the expected waveshape is visible as, for example, for subjects S02, S05, and S07 (wet).

The ERP images provide an additional overview of the single trials behind the observed average ERP for each subject. A first look comparing dry and wet systems suggests that the dry EEG seems more susceptible to noise, which masks the individual ERP components on a single-trial basis and could also explain the higher amplitudes in the average ERPs. It is also possible to see that, for subjects S01 and S06 (both dry and wet), the ERP components are not as clearly visible (per trial) as, for example, for subjects S05 or S07. Overall, the positive peak around 300 ms seems to be generated in every trial with high amplitudes for most subjects (more clearly visible in the wet data). On the other hand, we consider that the other peaks are not as clear on a single-trial basis, showing the inter-subject and trial variabilities while they underwent the same experimental task. It seems possible that only some single trials contribute to the high peaks observed in the average ERP, highlighting the relevance of further investigating the error-related on a single-trial basis instead of relying only on the averages over all trials and subjects.

These variabilities could also be responsible for the differences in the classification accuracies of both datasets. When comparing the subject-specific classifiers, the one-sided Wilcoxon signed-rank test showed that the TPR (percentage of error trials correctly classified) was significantly better (*p* = 0.039) for the classifier trained using the wet EEG data compared to the dry EEG data. The generic classifier also performed significantly better in the wet EEG dataset, but only for the TNR [percentage of correct trials correctly classified, *p* = 0.008, one-sided test for generic (wet) better than generic (dry)].

The ErrP waveshape also seems to vary across error types and even similar tasks in terms of latencies, amplitudes, and components (Xavier Fidêncio et al., 2022). How the experimental task, recording device, error rate, and feedback type, not to mention subject-related aspects such as anatomy, motivation, attention, and fatigue, affect the generated ErrP has to be better quantified. The more we know about the generation and overall waveshape characteristics of the ErrP signal, the more realistically it can be simulated. Our expectation is still if the simulated data represents the ErrP activity in a very realistic way, then a generic classifier trained only in the simulated data should perform at least as well as a classifier trained on the data of a pool of real-subjects.

Secondly, the simulation can be improved by including other aspects of EEG recordings. As other works using the toolbox SERREGA have already shown, it is possible to simulate the subject's habituation to the stimulus and fatigue (Krol et al., 2018), electrode shifts (Pawlitzki et al., 2022), or electrode pops, drifts, and noise (Kobler et al., 2019; Kumaravel et al., 2022; Sujatha Ravindran and Contreras-Vidal, 2023). Common EEG artifacts such as eye blink and muscle activity could also be included using a head model that also has ocular and muscular sources, such as the HArtMuT (Harmening et al., 2022). It is also possible to mix signal and noise at different signal-to-noise ratios (SNR). How the generic classifier accuracy is affected based on the chosen SNR will also be systematically investigated in future works.

One could also argue whether a simple SVM classifier is the best option as a generic classifier. Alongside LDA, this model is widely used for ErrPs classification (Yasemin et al., 2023) and serves as a good baseline for a proof-of-concept study. Recent works have focused on the application of more complex classification models for ErrP detection, mostly based on convolutional neural networks (Behncke et al., 2018; Swamy Bellary and Conrad, 2019; Gao et al., 2020). Interesting, however, is that little improvement is reported. For us, this is also an indicator that the ErrP classification accuracy is not only dependent on the machine learning technique used. We believe that the conditions under which the error signals are generated need to be better understood to guide the precise specification of preprocessing and classification pipelines. We ask ourselves, what if it is also possible that, on a single-trial basis, a distinguishable error signal is not available at the scalp level? If this would be the case, classification accuracies would already be fundamentally limited.

Nonetheless, more complex models and other machine learning techniques could be further investigated. The generic model could be modified using adaptation techniques to incorporate subject-specific information and improve the classification accuracy, as shown in Schonleitner et al. (2019, 2020). The number of trials and subjects used for training should also be evaluated, as well as the error rate used for the data simulation. As mentioned by Schonleitner et al. (2019), training data selection based on suitable similarity criteria could also be investigated.

In summary, BCIs are usually designed for each subject and require a subject-specific classifier to decode the brain signals of the particular user. In BCIs that apply ErrPs to improve their performance, the implication is that more than one subject-specific classifier needs to be trained, increasing the calibration sessions. Existing works suggest that a generic ErrP classifier can be used to remove or at least reduce the calibration time for new subjects (see works reviewed in Section 2). However, a decrease in performance is still present when compared to baseline classifier trained for each subject. Therefore, the purpose of this work is to contribute toward a truly generic subject-independent ErrP classifier. For that, we propose and validate a new framework for training a generic ErrP classifier. Similar to the works reviewed, we apply an inter-subject, intra-ErrP-type-transfer. On the other hand, we propose using simulated subjects for the generic decoder training. Using simulated data frees subjects from a tedious task and allows more flexibility regarding the data to use for training. We hypothesize that if the simulated data covers the inter-subject variabilities underlying the ErrP generation, a generic classifier trained on this data will be able to generalize to unseen subjects with little or no loss in performance.

## Data availability statement

The datasets presented in this article are not readily available because the simulated datasets used for this study can be obtained from the corresponding author on a reasonable request. Requests to access the datasets should be directed at: AX (aline.xavierfidencio@rub.de).

## Ethics statement

The studies involving humans were approved by Ethics Committee of Medical Faculty of the Ruhr University Bochum. The studies were conducted in accordance with the local legislation and institutional requirements. The participants provided their written informed consent to participate in this study.

## Author contributions

AX: Conceptualization, Formal analysis, Investigation, Methodology, Software, Visualization, Writing – original draft, Writing – review & editing. CK: Conceptualization, Funding acquisition, Resources, Supervision, Writing – review & editing. II: Conceptualization, Funding acquisition, Resources, Supervision, Writing – review & editing.

## Figures and Tables

**Figure A1:**
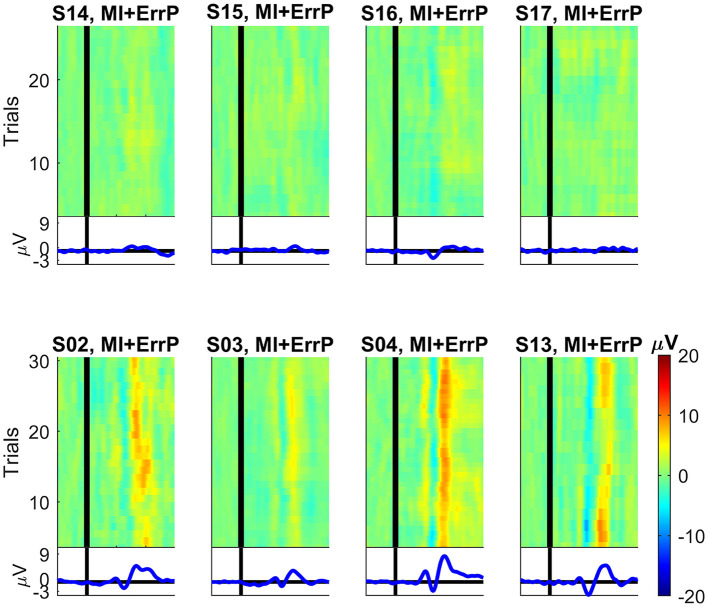
ERP images for the subjects recorded during the motor imagery and ErrP experiment at channel Cz (dataset 2). On the top we show the four rejected subjects and on the bottom four subjects for whom a clear ErrP signal is visible for comparison. For each subject, the average over all correct trials were subtracted from each error trial. After smoothing over five trials, the data was color-coded and combined into one image. First trial is at the bottom and last trial is at the top of each plot. For comparison, all plots have the same scale. The vertical black lines denote the time *t* = 0 ms (feedback onset).

## Footnotes

^1^SEREEGA is available under: https://github.com/lrkrol/SEREEGA.

## Appendix A

For four subjects recorded in the MI + ErrP experiment a prominent ErrP is also not clearly visible in the recorded data, as can be seen in Figure A1. As neither a loso nor the generic classifier performed well in the data of these four subjects for the error detection, they were removed from further analysis.
