# Supplementary material for: A generic error-related potential classifier based on simulated subjects
Source: Front Hum Neurosci. 2024 Jul 17;18:1390714. doi: 10.3389/fnhum.2024.1390714 (PMC11288877; doi:10.3389/fnhum.2024.1390714)
Supplement: Supplementary file 1 [file Data_Sheet_1.PDF]

Supplementary Material

1 EXPERIMENTAL PROTOCOL

1.1 EEG headset setup

For all recordings performed, we turned off all electronic devices in the room that were not mandatory for the recording itself to minimize electrical interference. For both EEG headsets, a proper cap size for the subject was chosen. The setup for each headset was executed as follows:

- Dry EEG system: as the impedance for dry electrodes is generally high, impedance measurements were not required. The dry EEG system allows adjusting the electrodes' pressure to improve signal quality by screwing them in or out or changing pin size to enhance scalp contact. A balance between signal quality and pressure was achieved for each individual to avoid discomfort or pain. Signal quality could sometimes be improved by applying circular motion, slight wiggling, or tilting the electrodes to remove hair between the electrode and scalp, as recommended by the manufacturer. For all subjects, we additionally used a cotton swab and a skin-friendly disinfectant to help remove hair between the electrode and scalp, which provided a quicker setup. Signal quality was determined by visual inspection of the EEG data, with consistent visualization parameters set for all assessments.
- Wet EEG system: we also used cotton swabs and disinfectant to help remove hair and expose the scalp before applying the conductive gel with a curved syringe. The EEG signal quality was assessed using the manufacturer's Quality Index (QI), since impedance checks for this headset are only available for stimulation channels. Each channel displayed a color code (red, orange, green) in the recording software. The manufacturer recommends starting recordings when the indicator shows green or orange. We only began recordings when all channels showed green, which was typically achieved immediately after filling the electrodes with sufficient conductive gel.

By following these procedures, we ensured optimal signal quality for each EEG system.

1.2 Real-life dataset 2 (motor imagery + ErrP)

The subjects played a modified version of the snake game shown in Figure 2 in the manuscript. In the version adapted for motor imagery-based control (see Supplementary Figure S1), instead of pressing a key on the keyboard, subjects were instructed to imagine the left or right-hand movement to control the snake. Chosen movements were either an open-hand gesture or squeezing a ball. To ensure a ground-truth for the motor imagination to be executed, a path from the snake to the fruit was shown and subjects were instructed to control the snake as to follow this path. Interaction errors were artificially introduced with a low rate of 5% to keep subjects motivated in this task, since the primary goal of this study was the collection of motor imagery data, not ErrPs. Motor imagery was not decoded online in this study. Subjects were told they were in charge of controlling the snake and should focus on improving motor imagination in case mistakes happened (error trials). They were unaware of the fact that the snake movement was programmed to follow the given path and that errors were artificially introduced. Subjects were allowed to play the game from some trials during familiarization phase. Data was recorded in a single session using the wet EEG headset and between $600 - 960$ trials were collected for each participant. The different number of trials is because subjects were allowed to interrupt the recording if they were no longer able to focus on the motor imagery task, which can be tiring.

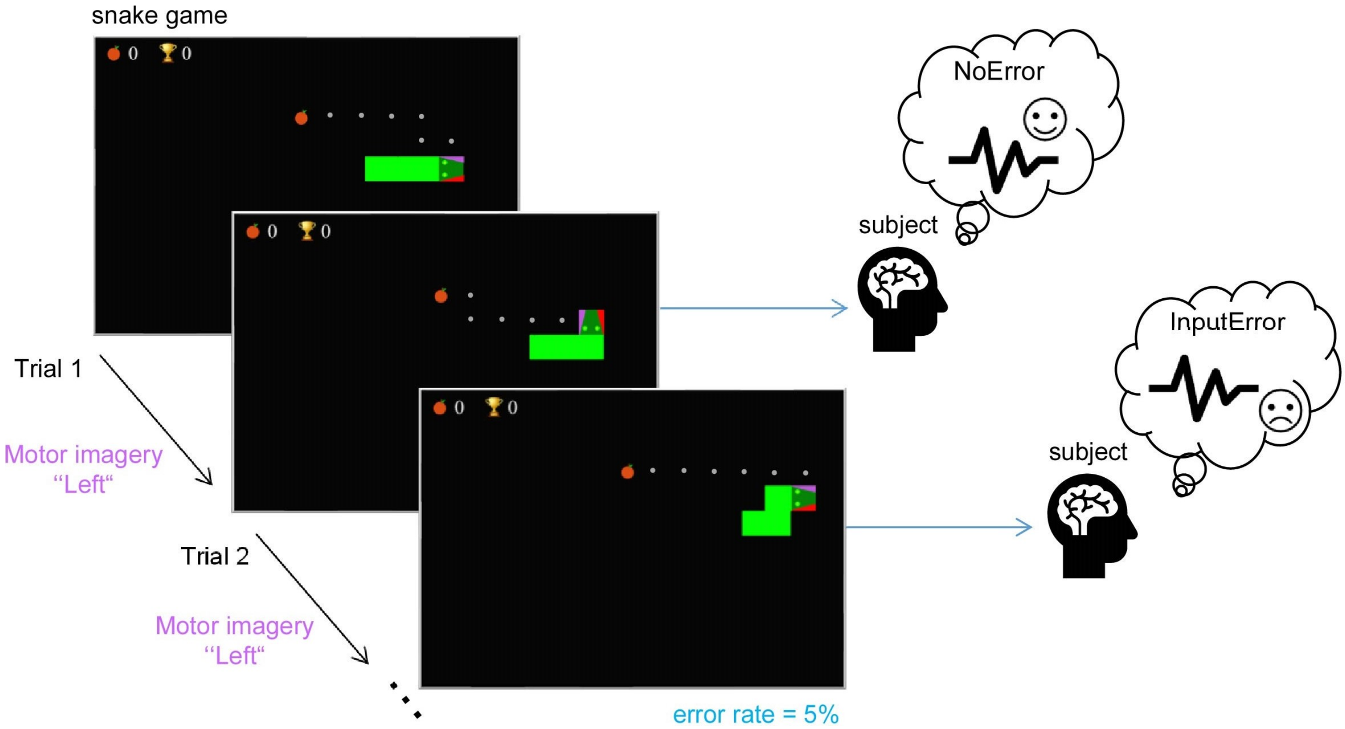

Figure S1. The experimental task used for the real-life data: the subject played the game by imagining either the left- or right-hand movement to control the snake's direction to avoid collision with itself, follow the given path (the displayed dots), and collect as many points as possible. With a probability of 5%, the snake moved in the wrong direction (as depicted in Trial 2) to keep subjects motivated, which also elicited an ErrP.
